# Carotid endarterectomy versus stenting in octogenarians

**DOI:** 10.1186/1471-2318-11-S1-A55

**Published:** 2011-08-24

**Authors:** A Savanelli, R Compagna, D De Vito, R Rossi, F Fappiano, T Bianco, M Amato, B Amato

**Affiliations:** 1 Dipartimento Assistenziale di Chirurgia Generale, Geriatrica ed Endoscopia Diagnostica ed Operativa. Università degli Studi di Napoli “Federico II”, Napoli, Italy

## Background

Stents are an alternative treatment to carotid endarterectomy (CEA) for symptomatic carotid stenosis, but previous trials have not established equivalent safety and efficacy in octuogenarian patients. We compared the safety of carotid stenting carotid endarterectomy (CAS). We conducted retrospective review of all cases of carotid stenosis treated with CEA and CAS.

## Materials and methods

During a 7 year period from October 2000 to April 2007, 84 symptomatic carotid stenosis were treated at Department of general surgery of AUO “Federico II” of Naples 48 with CAS and 36 with CEA. We divided our patients in two group not randomly but according to valuation anesthesia. The age range in this group was 80 to 89 years (average age of 82.7 years). The indication for treatment were transient ischemic attack in (43.1%). Cerebrovascular accident in (23%), amaurosis fugax in (9.3%), vascular tinnitus in (2.6%) Associated risk factors included systemic arterial hypertension, diabetes mellitus, coronary artery disease and significant smoking history. All procedures were performed under local anesthesia. Concomitantly or during the same hospitalization two patients underwent adjunctive procedures (coronary artery bypass, lung resection, colon resection).

## Results

Carotid endarterectomies in octogenarian patients represented 18% of the total carotid endarterectomies performed at AUO “Federico II” of Naples. The postoperative hospital stay averaged 5.4 days for CEA and 3.7 for CAS. Thirty-day morbidity and mortality included 6 (16.6%) death for CEA and 2 (4.1%) death for CAS. There were no postoperative strokes in the CEA group while there were four postoperative strokes in the CAS group. There were 6 cases (12.5%) of total complication in the CAS group and 4 cases (11.1%) in the CEA group. Long term follow-up results were available. at 3 years survival 30 (62%) in CAS group and 30 (79%) in CEA group.

## Conclusions

The incidence of stroke, death or procedural myocardial infarction was 12.5% in the stenting group compared with (11.1%) in the endarterectomy group. (CAS n°6 / CEA n°4). Risks of any stroke and all-cause of death were higher in stenting group than in the endarterectomy group. There were also fewer haematomas of any severity in the stenting group than in the endarterectomy group. Long-term follow-up is needed to establish the efficacy of carotid artery stenting compared with endarterectomy. In meantime, carotid endarterectomy should remain the treatment of choice for patients suitable for surgery. This experience suggests that carotid endarterectomy can be performed in elderly population with morbidity and mortality rates similar to those in a younger cohort. This suggests that if guidelines similar to those used in younger population are followed, paying close attention to associated risk factors, carotid endarterectomy can be performed safely in the elderly population. With the current trend toward growth of the aging population in our society, this information may become increasingly important for prevention of stroke and preservation of quality of life in a major segment of the population.
